# Personalizing voriconazole dosing in Chinese hematological patients: CYP2C19 phenotype and albumin-bilirubin grade as key predictors of trough concentrations

**DOI:** 10.3389/fphar.2025.1667461

**Published:** 2025-10-16

**Authors:** Lin Hu, Xi Tang, Yanfei Li, Juanjuan Huang

**Affiliations:** ^1^ Department of Pharmacy, The Affiliated Changsha Hospital of Xiangya School of Medicine, Central South University, Changsha, Hunan, China; ^2^ Department of Pharmacy, The First Hospital of Changsha, Changsha, Hunan, China; ^3^ Department of Pharmacy, Xiangya Hospital, Central South University, Changsha, Hunan, China

**Keywords:** voriconazole, CYP2C19 phenotype, albumin-bilirubin grade, hematological patients, therapeutic drug monitoring

## Abstract

**Purpose:**

This retrospective, single-center study aimed to evaluate the genetic and non-genetic factors influencing voriconazole (VRC) trough concentration (*C*_trough_), efficacy and safety in hematological patients.

**Methods:**

Medical records of inpatients were reviewed retrospectively. Univariate and multivariate analyses were performed to identify factors contributing to the variability of VRC *C*_trough_.

**Results:**

A total of 375 VRC *C*_trough_ measurements from 89 patients were analyzed. At the time of the initial *C*_trough_ assessment, 74 patients (83.1%) received oral VRC, while 15 patients (16.9%) received intravenous VRC. Among these first *C*_trough_ measurements, 68.5% of patients achieved the target therapeutic range (1.0–5.5 mg/L), whereas 28.1% had subtherapeutic concentrations and 3.4% had supratherapeutic concentrations. The dose-normalized VRC *C*_trough_ (*C*_trough_/D) were significantly higher in poor metabolizers (PMs) compared to normal metabolizers (NMs) (*P* = 0.001) and intermediate metabolizers (IMs) (*P* = 0.021). The albumin-bilirubin (ALBI) grade, a novel liver function assessment tool, was significantly associated with VRC *C*_trough_/D. Patients with ALBI grade 3 had significantly higher *C*_trough_/D values compared to those with grade 2 (*P* = 0.001) and grade 1 (*P* < 0.001). The linear mixed model revealed that sex, concomitant glucocorticoid use, creatinine clearance rate (Ccr), CYP2C19 genotype, and ALBI grade were statistically significant predictors of VRC *C*_trough_/D. A total of 10 patients (11.2%) had their VRC dosage adjusted based on therapeutic drug monitoring (TDM). The overall treatment success rate was 75.3% (67/89). Adverse drug reactions (ADRs) were observed in 12 patients (13.5%) during VRC therapy.

**Conclusion:**

CYP2C19 phenotype, ALBI grade, sex, Ccr and concomitant use of glucocorticoids contribute to the variability of VRC *C*_trough_ and should be comprehensively considered when determining VRC dosage in Chinese hematological patients.

## Introduction

Invasive fungal infections (IFIs) are opportunistic infections with high mortality rates that occur primarily in immunocompromised patients, especially in those with hematological diseases (Wang et al., 2019). Voriconazole (VRC), a broad-spectrum triazole antifungal agent, is the first-line treatment for the prevention and management of IFIs (Patterson et al., 2016). Given its nonlinear pharmacokinetics and high inter- and intra-individual variability, therapeutic drug monitoring (TDM) is essential. Adverse drug reactions (ADRs) associated with VRC, including hepatotoxicity, visual disturbances, and hallucinations, have been correlated with elevated trough concentrations (*C*_trough_) (Hanai et al., 2021; Jin et al., 2016). Therefore, individualized VRC dosing is critical to optimizing therapeutic efficacy while minimizing the risk of toxicity.

VRC is primarily metabolized in the liver by the cytochrome P450 2C19 (CYP2C19) enzyme (Weiss et al., 2009). CYP2C19 exhibits genetic polymorphisms, with allelic variants such as *2, *3, and *17 contributing to interindividual differences in metabolic capacity. According to the Clinical Pharmacogenetics Implementation Consortium (CPIC) guidelines (CPIC, 2018), the distribution of CYP2C19 phenotypes varies between Asian and Caucasian populations, potentially affecting optimal VRC dosing. The 2022 Japanese Society of Chemotherapy and the Japanese Society of Therapeutic Drug Monitoring (JSC/JSTDM) consensus (Takesue et al., 2022) recommends population-specific VRC dosing strategies for Asians and non-Asians to minimize the risk of overdose. Beyond genetic variation, our previous research has also identified non-genetic factors—including age, concomitant medications, and liver function, as significant contributors to variability in VRC *C*_trough_ (Hu et al., 2024a).

Our recent research demonstrated a significant correlation between VRC *C*_trough_ and Child-Pugh (CP) classification in patients with hepatic dysfunction (Hu et al., 2024b). Similarly, a population pharmacokinetic (PPK) study by Tang et al. identified a significant association between VRC clearance (CL) and total bilirubin (TBIL) levels (Tang et al., 2021). In clinical practice, VRC dosing is frequently adjusted based on the patient’s liver function. While the CP classification remains the most commonly used tool for liver function assessment, recent studies (Nashimoto et al., 2023; Asai et al., 2025) have suggested that the albumin-bilirubin (ALBI) grade may also correlate with VRC *C*_trough_ and could aid in optimizing initial dosing and predicting hepatotoxicity. The ALBI grade is calculated using two readily accessible biomarkers—serum albumin (ALB) and TBIL, both of which are obtained through routine blood tests.

Therefore, this study retrospectively collected clinical data from patients who had simultaneous measurements of steady-state VRC *C*_trough_ and CYP2C19 genotypes. Liver function was assessed using the ALBI grade. The primary objective of this study was to evaluate the influence of both genetic and non-genetic factors on VRC *C*_trough_. In addition, the study assessed the efficacy and safety of VRC in Chinese hematological patients, providing a scientific basis for the individualized use of VRC in this population.

## Methods

### Study design

We retrospectively reviewed the medical records of inpatients who had received VRC and undergone measurement of steady-state VRC *C*_trough_ and CYP2C19 genotyping through the department of hematology at Xiangya Hospital of the Central South University between 01 May 2015 and 01 May 2021. Inclusion criteria: (i) patients aged ≥15 years. (ii) patients who underwent measurement of steady-state VRC plasma *C*_trough_ and CYP2C19 genotyping during hospitalization. Exclusion criteria: patients who received concomitant antifungal agents in addition to VRC. Steady-state was considered to be reached at 24 h following oral or intravenous loading dose and regarding the evidence and variability among patients, obtaining the first blood sample on day 3. Without oral or intravenous loading dose, it was reported that steady-state was considered to be reached on day 4–7 of twice daily dosing (Chen et al., 2018).

### Ethics

This retrospective study strictly followed the Helsinki Declaration and the protocol was approved by the Institutional Review Board of Xiangya Hospital (Approval number 2018091069). The identity information of all patients in this study has been coded to ensure that identity information is not leaked. The data are anonymous, and the requirement for informed consent was therefore waived.

### Measurement of VRC plasma *C*_trough_ and CYP2C19 phenotype

All *C*_trough_ were collected 30 min before the next dose. The measurement of VRC *C*_trough_ was performed by the methods described in our previous publication (Hu et al., 2018). In brief, analysis of VRC concentrations was performed using high-performance liquid chromatography (analytical range, 0.02–19.60 mg/L). According to our previous experience, the target range of VRC *C*_trough_ is still 1.0–5.5 mg/L in this study (Hu et al., 2018). Each patient could have multiple steady-state *C*_trough_ measurements during hospitalization. Detection of CYP2C19 *2, *3, and *17 alleles, associated with reduced CYP2C19 enzymatic activity, was performed using a DNA microarray chip method (BaiO^®^, Shanghai, China). Based on genotyping results, patients were categorized as follows: ultra-rapid metabolizers (UM, *17/*17), rapid metabolizers (RM, *1/*17), normal metabolizers (NM, *1/*1), intermediate metabolizers (IM, *1/*2, *1/*3, *2/*17, or *3/*17), and poor metabolizers (PM, *2/*2, *2/*3, or *3/*3).

### Data collection

Medical records were retrieved from the electronic medical record system. The following patient data were extracted: ethnicity, diagnosis of IFI, treatment indication, dosage and duration of VRC therapy, route of administration, concomitant medications, VRC TDM results, CYP2C19 phenotype, ALB, TBIL, and serum creatinine (Scr). The ALBI grade was calculated using the formula:

$$\text{ALBI}=\left(0.66\times \log_{10}\left[\text{TBIL},\mu \text{mol}/L\right]\right)\;‐\;\left(0.085\times \text{ALB }\left[g/L\right]\right)$$

ALBI grades were categorized as follows: grade 1 (≤−2.60), grade 2 (−2.59 to −1.39), and grade 3 (>−1.39) (Asai et al., 2025), with higher scores indicating poorer liver function. Renal function was assessed using the creatinine clearance rate (Ccr).

### Evaluation of efficacy and safety

Treatment response was evaluated based on the updated guidelines of the European Organization for Research and Treatment of Cancer/Invasive Fungal Infections Cooperative Group and the National Institute of Allergy and Infectious Diseases Mycoses Study Group (EORTC/MSG) (Donnelly et al., 2020). IFIs were categorized as possible, probable, or proven according to the EORTC/MSG criteria. ADRs were assessed using the National Cancer Institute’s Common Terminology Criteria for Adverse Events (CTCAE), version 5.0 (CTCAE). A VRC-related ADR was defined as one with possible or stronger relationship.

### Statistical analysis

All statistical analyses were conducted using SPSS version 25.0. Univariate and multivariate analyses were performed to identify factors associated with VRC *C*_trough_. To account for variability in dosage and body weight, the standardized dose normalized to body weight - *C*_trough_ (mg/L)/D (mg/kg) was utilized. Variables included in the univariate analysis were age, sex, route of administration, concomitant use of glucocorticoids and proton pump inhibitors (PPIs), Ccr, CYP2C19 genotype, and ALBI grade. The *χ* ^2^ test or Fisher’s exact test was used to compare categorical variables, while the Mann–Whitney U test and Kruskal–Wallis test were applied for continuous variables. Spearman’s correlation coefficient was employed to assess the associations between continuous variables. A linear mixed model was applied for the multivariate analysis of factors influencing VRC *C*_trough_. The dependent variable was defined as log(*C*_trough_/D). To address the non-independence of repeated measurements within the same patient, patient ID was included as a random intercept. Fixed effects were selected based on variables identified in the univariate analysis. Each CYP2C19 polymorphism was evaluated for compliance with Hardy–Weinberg equilibrium. A two-sided *P*-value < 0.05 was considered statistically significant.

## Results

### Patient characteristics

A total of 168 patients were initially screened. After excluding those < 15 years (*n =* 50), those lacking CYP2C19 genotype data (*n =* 19), and those without steady-state VRC *C*_trough_ (*n =* 10), 89 patients remained for the final analysis. Fifth-five (61.8%) were male, 34 (38.2%) were female. The median age and weight were 33 years old (range, 15–68 years old) and 55 kg (range, 41–83 kg), respectively. All patients had malignant hematological diseases. Thirty-six (40.5%) of patients had acute myeloid leukemia. Proven, probable, and possible IFIs were reported for 5 (5.6%), 20 (22.5%), and 64 (71.9%) patients, respectively. Sixty-six (74.2%) patients received a coadministration of VRC and PPIs, and 47 (52.8%) patients received a coadministration of VRC and glucocorticoids. Patient characteristics are summarized in Table 1.

**TABLE 1:** Patient characteristics (*n* = 89).

Parameters	Value^a^
Age (years), median [range]	33 (15–68)
Body weight (kg), median [range]	55 (41–83)
Sex (male)	55 (61.8)
Ethnicity, Asian	89 (100)
Neutrophil deficiency	54 (60.7)
CYP2C19 phenotype	
NM	41 (46.1)
IM	36 (40.4)
PM	12 (13.5)
IFI diagnosis	
Proven	5 (5.6)
Probable	20 (22.5)
Possible	64 (71.9)
Underlying conditions	
Acute myeloid leukemia	36 (40.5)
Acute lymphoblastic leukemia	26 (29.2)
Myelodysplastic syndrome	10 (11.2)
Aplastic anemia	8 (9.0)
Lymphoma	6 (6.7)
Chronic myelogenous leukemia	3 (3.4)
Administration routes	
Oral	74 (83.1)
Intravenous	15 (16.9)
Concomitant medications	
PPIs^b^	66 (74.2)
Glucocorticoids^c^	47 (52.8)
Reason for VRC use	
First line use	56 (62.9)
Failure of other antifungal agent	33 (37.1)
ALBI grade	
Grade 1	12 (13.5)
Grade 2	71 (79.8)
Grade 3	6 (6.7)

NM, normal metabolizer; IM, intermediate metabolizer; PM, poor metabolizer; IFI, invasive fungal infection; PPIs, proton pump inhibitors; VRC, voriconazole; ALBI, albumin-bilirubin.

^a^ Values are number of patients (percent) unless otherwise indicated.

^b^ The PPIs, used were pantoprazole (*n* = 27), omeprazole (*n* = 26) and lansoprazole (*n* = 13).

^c^ The glucocorticoids used were methylprednisolone (*n* = 26), dexamethasone (*n* = 19) and prednisone (*n* = 2).

### VRC dosing and *C*_trough_

**FIGURE 1:**
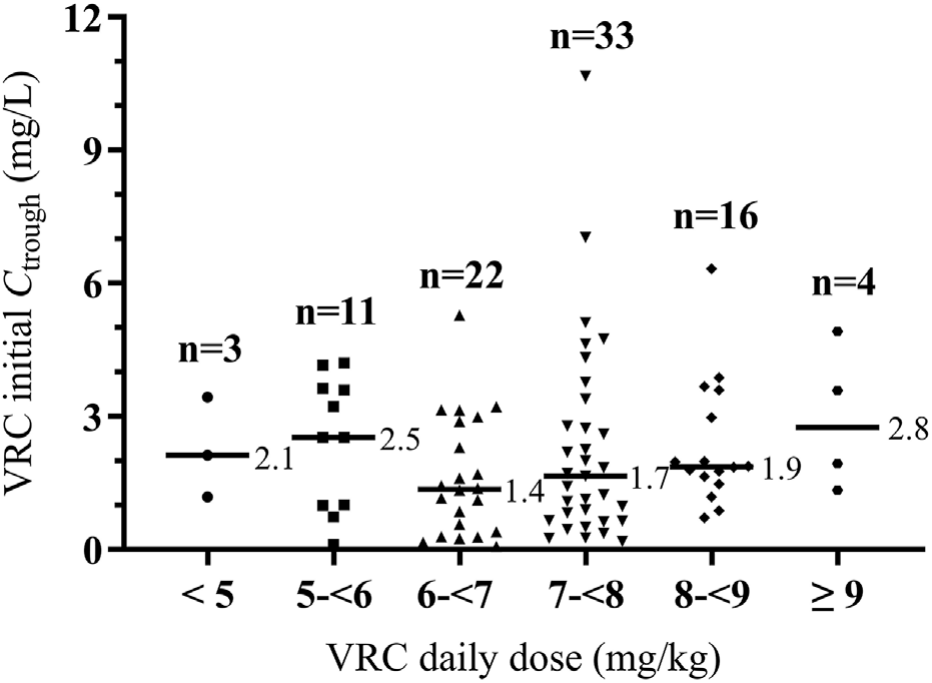
Distribution of initial VRC *C*_trough_ across different weight-adjusted dosing groups. Horizontal bars represent median initial trough value for each dose group. VRC, voriconazole. *C*_trough_, trough concentration.

A total of 375 VRC *C*_trough_ were measured from 89 patients in this study. The median number of measurements per patient was 3 (range, 1–20). The median VRC initial *C*_trough_ was 1.76 mg/L (range, 0.06–10.67 mg/L). At the measurement of the first *C*_trough_, the target range was achieved in 68.5% of patients, while subtherapeutic and supratherapeutic concentrations were obtained in 28.1% and 3.4% of patients, respectively. The median duration of VRC treatment was 24 days (range, 7–163 days). At the time of the initial *C*_trough_ measurement, 74 patients (83.1%) received oral VRC, while 15 patients (16.9%) received intravenous VRC. The median intravenous and oral maintenance daily dose to reach a therapeutic range was 7.3 mg/kg (range, 4.8–8.3 mg/kg) and 7.1 mg/kg (range, 4.6–9.8 mg/kg), both significantly lower than the recommended dose of 8 mg/kg (*P* = 0.009 and *P* < 0.001, respectively). The distribution of initial VRC *C*_trough_ values across different weight-adjusted dose groups is shown in Figure 1.

### Univariate analysis to explore factors affecting VRC *C*_trough_

**FIGURE 2:**
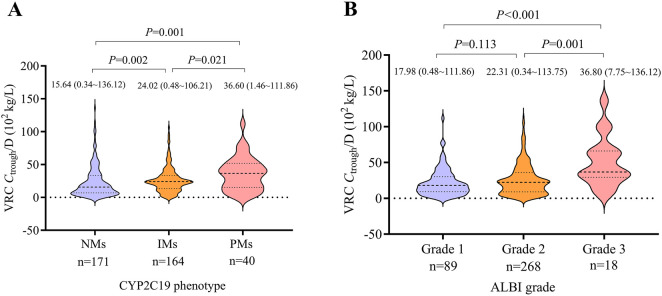
Violin plots illustrating the distribution of *C*_trough_/D across different CYP2C19 phenotypes and ALBI grades. **(A)** Comparison of VRC *C*_trough_/D among CYP2C19 phenotypes. **(B)** Comparison of VRC *C*_trough_/D across ALBI grades. Median values (with ranges) and corresponding *P*-values are indicated above each plot. The number of VRC *C*_trough_ measurements in each group is displayed below the X-axis. VRC, voriconazole. *C*_trough_/D, trough concentration to dose ratio. NM, normal metabolizer. IM, intermediate metabolizer. PM, poor metabolizer. ALBI, albumin-bilirubin.

The wild-type CYP2C19 phenotype (NM) was the most commonly identified phenotype (41/89 patients [46.1%]), followed by the mutant types IM (36/89 patients [40.4%]) and PM (12/89 patients [13.5%]). No UMs or RMs were identified in this study. The allele frequencies of the CYP2C19*2 and CYP2C19*3 alleles were 30.3% and 3.4%, respectively. The Hardy-Weinberg equilibrium was respected for each allele (CYP2C19*2, *χ* ^*2*^ = 0.009, *P* = 0.92; CYP2C19*3, *χ* ^*2*^ = 0.11, *P* = 0.74). A significant difference in *C*_trough_/D was observed among the three CYP2C19 phenotypes. PMs exhibited significantly higher *C*_trough_/D values compared to NMs and IMs (*P* = 0.001 and *P* = 0.021, respectively). Additionally, IMs had significantly higher *C*_trough_/D values than NMs (*P* = 0.002). At the time of the first *C*_trough_ measurement, subtherapeutic concentrations were observed in 46.3% (19/41) of NMs and 16.7% (6/36) of IMs. The comparison of VRC *C*_trough_/D across CYP2C19 phenotypes is presented in Figure 2A.

There were 12, 71, and 6 patients with ALBI grades 1, 2, and 3, respectively. Patients with ALBI grade 3 showed significantly higher *C*_trough_/D values compared to those with grade 2 and grade 1 (*P* = 0.001 and *P* < 0.001, respectively). No significant difference was observed between ALBI grades 1 and 2. The comparison of VRC *C*_trough_/D across different ALBI grades is shown in Figure 2B.

The median *C*_trough_/D in male patients was 27.04 (range, 0.34–136.12), which was significantly higher than in female patients, whose median *C*_trough_/D was 15.21 (range, 0.38–96.66) (*P* < 0.001). Patients receiving concomitant glucocorticoids had a significantly lower median *C*_trough_/D of 18.82 (range, 0.34–113.75) compared to 24.75 (range, 0.48–136.12) in those not receiving glucocorticoids (*P* < 0.001). Additionally, patients receiving intravenous VRC had a significantly higher median *C*_trough_/D of 26.33 (range, 0.93–106.21) than those receiving oral administration, whose median was 20.73 (range, 0.34–136.12) (*P* = 0.026). Spearman’s correlation analysis indicated that *C*_trough_/D was positively associated with age (r = 0.103, *P* = 0.047) and negatively associated with Ccr (r = −0.127, *P* = 0.014). The median VRC *C*_trough_/D was 19.30 (range, 0.60–113.75) in patients ≤18 years and 21.46 (range, 0.34–136.12) in patients >18 years, with no significant difference between the two groups (*P* = 0.896). No significant difference in *C*_trough_/D was observed between patients with and without concomitant use of PPIs (*P* = 0.098).

### Multivariate analysis by linear mixed model

Significant variables identified in the univariate analysis were included in the linear mixed model, comprising seven factors: age, sex, route of administration, concomitant use of glucocorticoids, Ccr, CYP2C19 genotype, and ALBI grade. The results indicated that sex, concomitant use of glucocorticoids, Ccr, CYP2C19 genotype, and ALBI grade had a statistically significant impact on VRC *C*_trough_/D. No significant associations were observed for the remaining covariates, as presented in Table 2.

**TABLE 2:** The results of linear mixed model for factors affecting the VRC *C*_trough_/D.

Variable	β (95% CI)	*P* value
Age (years)	0.002 (−0.003, 0.008)	0.430
Male^a^	0.194 (0.053, 0.334)	0.008
Oral administration^b^	−0.099 (−0.287, 0.089)	0.298
Ccr (mL/min)	−0.003 (−0.005, −0.001)	0.002
Concomitant use of glucocorticoids^c^	0.108 (0.011, 0.204)	0.028
CYP2C19 phenotype^d^		
IM	−0.166 (−0.306, −0.026)	0.021
PM	−0.233 (−0.446, −0.020)	0.032
ALBI grade^e^		
Grade 2	−0.033 (−0.155, −0.088)	0.591
Grade 3	−0.570 (−0.812, −0.328)	0.000

VRC, voriconazole. *C*_trough_/D, trough concentration to dose ratio. Ccr, creatinine clearance rate. IM, intermediate metabolizer; PM, poor metabolizer; ALBI, albumin-bilirubin; CI, confidence interval.

^a^ Compared to female.

^b^ Compared to intravenous administration.

^c^ Compared to patients without concomitant use of glucocorticoids.

^d^ Compared to normal metabolizer.

^e^ Compared to albumin-bilirubin grade 1.

### Dose adjustments based on TDM

A total of 10 patients (11.2%) adjusted dose according to TDM during VRC treatment. Six patients with low concentration of VRC or lack of response increased the VRC dose (25% dose increase in one patient, 33.3% dose increase in three patients, and 50% dose increase in two patients). Finally, VRC concentrations were elevated in all six patients after dose adjustments and five patients reached the target range. Four patients with high *C*_trough_ (>5.0 mg/L) or documented toxicity decreased the VRC dose (25% dose decrease in one patient, 33.3% dose decrease in one patient, 50% dose decrease in one patient, and discontinuance of VRC in one patient) and *C*_trough_ were decreased in all four patients.

### Efficacy and safety of VRC treatment

According to the criteria for therapeutic efficacy, the overall rate of the treatment success was 75.3% (67/89). The lack of response to VRC therapy was more frequent in patients with a VRC initial *C*_trough_ of < 1.0 mg/L (40.0%) than in patients with a VRC initial *C*_trough_ of ≥ 1.0 mg/L (18.8%) (*P* = 0.037). ADRs were observed in 12 patients (13.5%) during VRC treatment, including hepatotoxicity in six cases, hallucinations in two cases, diarrhea in two cases, unconscious in two cases and tremor in one case. Only one patient with both unconscious and hepatotoxicity had an average VRC *C*_trough_ of 6.16 mg/L (>5.5 mg/L), and the remaining 11 patients had an average VRC *C*_trough_ of 0.73–3.29 mg/L. The average VRC *C*_trough_ in patients with documented toxicity was higher than patients without ADR (mean *C*_trough_, 2.40 mg/L vs. 1.91 mg/L, respectively, *P* = 0.195), however, it trended higher but was not significant. The effective rates for proven, probable, and possible IFIs were 60.0% (3/5), 65.0% (13/20), and 79.7% (51/64), respectively. No significant differences in efficacy were observed among the three groups (*P* = 0.297). The rates of ADRs were 40.0% (2/5), 20.0% (4/20), and 9.4% (6/64), respectively, with no statistically significant differences observed among the groups (*P* = 0.097).

## Discussion

This study evaluated the impact of both CYP2C19 genetic polymorphisms and non-genetic factors, including ALBI grade, on VRC *C*_trough_ in Chinese patients. It also investigated the VRC dosing required to achieve the target therapeutic range and described dose adjustments, efficacy, and safety within the study population.

Significant differences in VRC *C*_trough_ were observed among the three CYP2C19 phenotypes. In this study, PMs exhibited significantly higher VRC *C*_trough_ than NMs and IMs, were consistent with our previous studies (Hu et al., 2023a; Hu et al., 2023b). CYP2C19 phenotype has been shown to effectively guide initial VRC dosing and is often used to explain subtherapeutic concentrations (Zonios et al., 2014). NMs are less likely to present with subtherapeutic VRC levels. Our study demonstrated that patients with VRC concentrations < 1.0 mg/L exhibited poor treatment responses, aligning with findings by Pascual et al., who also reported that low VRC levels were associated with reduced therapeutic efficacy (Pascual et al., 2008). Treatment success significantly improves when the *C*_trough_ is ≥ 1.0 mg/L (Hanai et al., 2021). Therefore, the VRC dose in NMs should be appropriately increased to enhance the likelihood of achieving therapeutic levels and improving treatment efficacy. The CPIC guidelines (CPIC, 2018) recommended using CYP2C19 genotyping to inform VRC dosing strategies. Standard dosing is advised for NMs and IMs. For PMs, alternative antifungal agents not primarily metabolized by CYP2C19 are preferred. However, if VRC remains the most appropriate option, a reduced dose in combination with TDM is recommended.

Numerous pharmacogenomic studies have demonstrated that the CYP2C19 PMs has the highest prevalence in East Asian populations (approximately 13%–23%), which is substantially higher than in Caucasian populations (approximately 1%–6%) (Desta et al., 2002; Zhou et al., 2019; Lamoureux et al., 2016; Zonios et al., 2014). In our study, CYP2C19 PMs accounted for 13.5% of the cohort, a frequency consistent with previous reports. Consequently, patients with hematologic diseases in Asian populations may have an inherently higher risk of VRC overexposure and related toxicity compared with populations where the PMs is less frequent. Given the higher prevalence of PMs in Asian populations, a reduced maintenance dose may be necessary to attain comparable *C*_trough_. Due to genetic differences, the manufacturer’s original dosing recommendations (Pfizer Limited, 2012) may not be optimal for Chinese patients. The 2022 JSC/JSTDM Consensus (Takesue et al., 2022) recommended a maintenance dose of 3 mg/kg for Asian patients, considering the tendency for elevated *C*_trough_ levels and a higher incidence of ADRs.

Our findings underscore not only the universal importance of individualized VRC therapy but also its particular relevance in Asian populations. When extrapolating our results to other ethnic groups, the distribution of CYP2C19 genotypes in the target population must be carefully considered. For instance, in populations where the PMs is rare, other factors, such as drug–drug interactions and hepatic function, may play a comparatively greater role. Conversely, our data provide region-specific evidence supporting the implementation of CYP2C19 genotype-guided dosing strategies in Asian medical centers. Future multicenter, multiethnic studies are warranted to establish more generalizable models that can accurately quantify the combined effects of genotype, ethnicity, and clinical factors on VRC pharmacokinetics.

In this study, only CYP2C19 genetic polymorphisms were analyzed, while other enzymes and transporters that may also affect VRC metabolism were not evaluated. He et al. provided novel insights into the role of CYP3A4 in VRC pharmacokinetics (He et al., 2015). Similarly, Gautier-Veyret et al. (2016) demonstrated that a combined genetic score incorporating CYP2C19 and CYP3A4 genotypes could predict VRC *C*_trough_. However, other studies have reported inconsistent findings. For instance, no significant associations were observed between CYP3A4, ABCB1, or FMO3 genotypes and plasma VRC *C*_trough_ (Chuwongwattana et al., 2020). Furthermore, while several studies recommended CYP2C19 genotyping to optimize VRC dosing in patients, research in healthy Chinese adults suggested that polymorphisms in CYP2C9, CYP3A4, and FMO3 may have minimal impact on VRC pharmacokinetics (Liu et al., 2024).

Li et al. (2017) demonstrated that liver function and CYP2C19 polymorphisms are major determinants of VRC pharmacokinetic variability, a conclusion consistent with our findings. In our previous research, we also identified that ALB, alanine aminotransferase, and direct bilirubin levels significantly influence VRC *C*_trough_ (Hu et al., 2024b; Hu et al., 2023b). Accordingly, in this study, we adopted the ALBI grade to assess liver function instead of the CP classification. Unlike the CP system, which incorporates subjective factors such as ascites and hepatic encephalopathy, the ALBI grade offers a more objective and precise evaluation of liver function and is applicable to patients with both cirrhotic and non-cirrhotic liver diseases. Therefore, using ALBI grading to assess liver function for guiding VRC dosing may offer a more broadly applicable alternative to the CP classification. The recent PPK study of VRC in patients with hepatic dysfunction evaluated optimal dosing based on ALBI grading. The recommended regimens were 100 mg twice daily, 75 mg twice daily, and 50 mg twice daily for ALBI scores of −3, −2, and −1, respectively (Nashimoto et al., 2025).

Our previously published studies (Hu et al., 2018) demonstrated that VRC *C*_trough_ following intravenous administration were significantly higher than those observed after oral administration in pediatric patients. Similar findings were observed in the present cohort. A potential explanation is that VRC absorption differs between children and adults. The oral bioavailability of VRC in children is approximately 44% (Purkins et al., 2002), compared to over 90% in adults (Veringa et al., 2017) (the present study included patients as young as 15 years). Additionally, oral administration is more susceptible to first-pass metabolism and drug–drug interactions. Intestinal CYP3A4 also serves as a barrier to VRC absorption. Moreover, oral VRC absorption may be influenced by factors such as concurrent food intake, gastrointestinal complications, and diarrhea.

Allegra et al. (2018) reported that males had significantly higher median VRC *C*_trough_ than females and that *C*_trough_ was positively correlated with age. Similarly, Hashemizadeh et al. (2017) found that reduced VRC concentrations were associated with the concomitant use of glucocorticoids. These findings are consistent with the results of our study. In this study, 16.9% of patients received VRC via intravenous administration. The intravenous formulation contains sulfobutyl ether-β-cyclodextrin (SBECD), which is normally eliminated through the kidneys but may accumulate in patients with renal impairment, potentially leading to nephrotoxicity. At high cumulative doses (≥400 mg/kg), SBECD accumulation may further exacerbate renal dysfunction (Yasu et al., 2018). Dolton et al. (2014) proposed that glucocorticoids may induce cytochrome P450 enzymes, particularly CYP enzymes, thereby enhancing VRC metabolism and reducing its plasma concentration. Several studies have also examined the impact of PPIs on VRC *C*_trough_. For instance, Yan et al. (2018) reported that co-administration of omeprazole significantly increased VRC plasma levels. Hashemizadeh et al. (2017) similarly observed elevated VRC concentrations with the co-administration of omeprazole or pantoprazole. However, in our study, the concomitant use of PPIs did not have a statistically significant effect on VRC *C*_trough_. A possible explanation is that most patients in our cohort were co-administered pantoprazole or lansoprazole, which exert less inhibitory effect on CYP2C19 compared to omeprazole.

Although CYP2C19 polymorphisms significantly influence VRC *C*_trough_, relying solely on CYP2C19 genotyping is insufficient for accurately guiding VRC dose optimization. Therefore, it is essential to integrate nonlinear pharmacokinetics, CYP2C19 genotyping, and other non-genetic factors when determining initial VRC dosing. Dose adjustments should subsequently be guided by TDM. Significant non-genetic factors identified in this study, such as ALBI grade and concomitant use of glucocorticoids, Ccr should be considered in future prospective PPK studies. Only 10 patients in our study underwent dose adjustments based on TDM, which limits the strength of conclusions regarding the effectiveness of TDM-guided interventions. However, evidence from previous studies suggested that earlier implementation of TDM may improve outcomes. For instance, Shen et al. (2022) reported that VRC-induced hepatotoxicity occurred in 66.7% of patients within 7 days of the first dose and in 94.4% within 15 days. Hu et al. (2024b) reported that the median time to onset of ADRs after initiating VRC therapy was 7.5 days. These findings underscore the potential of early TDM to substantially improve clinical outcomes. The Chinese Pharmacological Society (CPS) guidelines (Chen et al., 2018) recommended increasing the maintenance dose of VRC by 50% if *C*_trough_ is < 1.0 mg/L or if treatment efficacy is inadequate. If the *C*_trough_ is between 5 and 10 mg/L without CTCAE grade 2 or higher adverse events, a 20% dose reduction is advised. If the *C*_trough_ > 10 mg/L or if CTCAE grade 2 or higher adverse events occur, VRC administration should be interrupted, followed by a 50% reduction in the maintenance dose upon resumption.

Recent researches have demonstrated that CRP, a biomarker of inflammation, is significantly correlated with VRC *C*_trough_ (Hu et al., 2025; Encalada Ventura et al., 2016). Inflammatory status may reduce VRC metabolism, leading to elevated VRC *C*_trough_. Specifically, for every 1 mg/L increased in CRP, VRC *C*_trough_ increased by approximately 0.015 mg/L (van Wanrooy et al., 2014). The risk of VRC overexposure and associated adverse reactions rised markedly in patients with CRP levels >102.23 mg/L (Lin et al., 2023). Furthermore, PPK studies have identified CRP as a significant covariate influencing the maximum enzymatic activity (V_max_) (van den Born et al., 2023). Consistently, Ling et al. (2024) reported that CRP is an important covariate affecting VRC clearance. However, our study did not include CRP concentration, preventing us from evaluating the impact of inflammatory factors on VRC *C*_trough_. Future large-scale prospective studies are warranted to investigate the optimal VRC dosing strategies based on CRP stratification.

The intrinsic characteristics of malignancy, such as disease type, stage, activity, and degree of inflammation, may indirectly affect VRC *C*_trough_ by altering systemic physiological conditions, including inflammatory cytokine levels, serum protein concentrations, and liver function. These factors may therefore serve as potential unmeasured confounders. However, due to the retrospective nature of our study, specific biomarkers and detailed baseline disease status could not be obtained. Malignancy status represents an incompletely measured confounding factor, which should be addressed in the design of future studies.

### Limitations

This study was conducted at a single institution using a retrospective design, which may introduce potential selection bias. Because all participants were recruited from one center, their demographic characteristics, underlying conditions, and prescribing practices may not fully represent a broader population, thereby limiting the generalizability of the findings. Moreover, retrospective studies often faced issues with incomplete data, such as genetic polymorphism of other metabolic enzymes (CYP3A4/5, FMO3). In addition, in our cohort, most patients did not have simultaneous measurements of VRC *C*_trough_ and CRP levels, limiting our ability to assess this relationship and potentially resulting in incomplete findings.

## Conclusion

CYP2C19 phenotype, ALBI grade, sex, Ccr and concomitant use of glucocorticoids were identified as significant contributors to the variability of VRC *C*_trough_/D and should be comprehensively considered when determining appropriate VRC dosing. Compared with the CP classification, ALBI grading may offer broader applicability for guiding individualized VRC therapy. Future PPK studies should incorporate these factors to establish more precise and personalized dosing strategies. Additionally, investigating VRC dose adjustment strategies in special populations, such as pediatric patients, individuals with hepatic impairment, or organ transplant recipients—is of particular importance due to limited existing data. Future research should prioritize these populations to enhance the efficacy and safety of VRC use.

## Data Availability

The original contributions presented in the study are included in the article/supplementary material, further inquiries can be directed to the corresponding authors.
